# Cardiac output monitoring in brain-stem-dead potential organ donors: an audit of current UK practice

**DOI:** 10.1186/cc12115

**Published:** 2013-03-19

**Authors:** CJ Wright, A Broderick, G Mandersloot

**Affiliations:** 1 Glasgow Royal Infirmary, Glasgow, UK; 2 National Health Service Blood and Transplant, London, UK; 3 The Royal London Hospital, London, UK

## Introduction

Significant changes in haemodynamics occur after brain stem death (BSD) and there is evidence that yield of transplantable organs may be decreased in donors who remain preload responsive prior to donation [1], suggesting that optimisation of the cardiac output (CO) may be beneficial in potential organ donors. We describe current UK practice with regard to CO monitoring in this group.

## Methods

We reviewed a database of 287 brain-stem-dead potential organ donors collected by specialist nurses in organ donation (SN-OD) over a 6-month period (30 April 2011 to 31 October 2011) across multiple UK centres. The database contained data on donor management in the period from initial SN-OD review to immediately prior to transfer to the operating theatre. We analysed data on CO monitoring and vasopressor/inotrope use. Where information was missing/not recorded in the dataset, the treatment referred to was interpreted as not given/not done.

## Results

**Figure 1:**
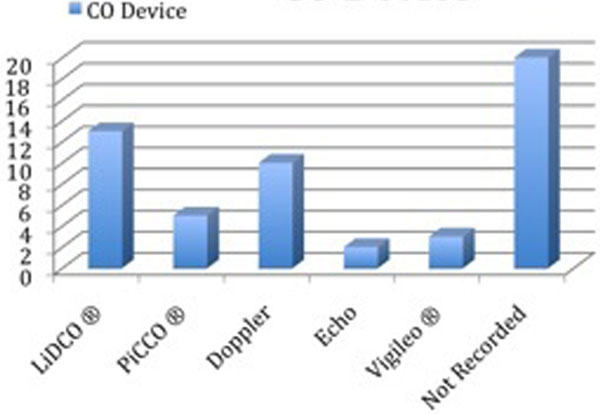
**CO device**.

**Figure 2:**
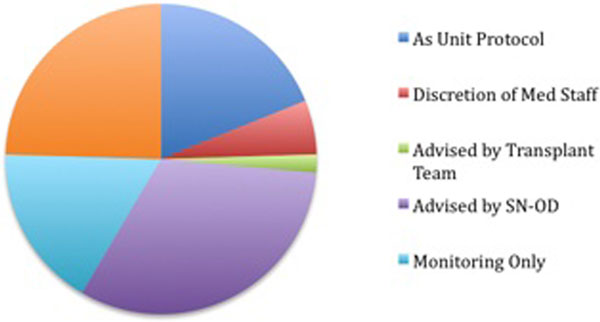
**Use of CO data**.

Fifty-three patients (18.5%) had evidence of CO monitoring. LiDCO was the most popular method (Figure 1). A total of 264 (94%) patients received treatment with vasopressors and/or inotropes. CO data were utilised in a variety of ways (Figure 2).

## Conclusion

The majority of potential donors require vasopressors and/or inotropes post BSD, but it seems only a minority currently have their CO monitored. There is variation in how CO data are utilised to direct haemodynamic management. We welcome the development of standardised bundle-driven donor management.
